# Autonomic nervous system monitoring in intensive care as a prognostic tool. Systematic review

**DOI:** 10.5935/0103-507X.20170072

**Published:** 2017

**Authors:** Luis Bento, Rui Fonseca-Pinto, Pedro Póvoa

**Affiliations:** 1 Medical Emergency Unit, Centro Hospitalar de Lisboa Central, EPE - Lisbon, Portugal.; 2 Instituto Politécnico de Leiria - Leiria, Portugal.; 3 Instituto de Telecomunicações, MSP - Leiria, Portugal.; 4 Multipurpose Intensive Care Unit, São Francisco Xavier Hospital - West Lisbon Hospital Center - Lisbon, Portugal.; 5 NOVA Medical School, CEDOC, Universidade Nova de Lisboa - Lisbon, Portugal.

**Keywords:** Autonomic nervous system, Heart rate variability, Intensive care, Prognosis, Sistema nervoso autônomo, Variabilidade da frequência cardíaca, Cuidados intensivos, Prognóstico

## Abstract

**Objective:**

To present a systematic review of the use of autonomic nervous system monitoring as a prognostic tool in intensive care units by assessing heart rate variability.

**Methods:**

Literature review of studies published until July 2016 listed in PubMed/Medline and conducted in intensive care units, on autonomic nervous system monitoring, via analysis of heart rate variability as a prognostic tool (mortality study). The following English terms were entered in the search field: ("autonomic nervous system" OR "heart rate variability") AND ("intensive care" OR "critical care" OR "emergency care" OR "ICU") AND ("prognosis" OR "prognoses" OR "mortality").

**Results:**

There was an increased likelihood of death in patients who had a decrease in heart rate variability as analyzed via heart rate variance, cardiac uncoupling, heart rate volatility, integer heart rate variability, standard deviation of NN intervals, root mean square of successive differences, total power, low frequency, very low frequency, low frequency/high frequency ratio, ratio of short-term to long-term fractal exponents, Shannon entropy, multiscale entropy and approximate entropy.

**Conclusion:**

In patients admitted to intensive care units, regardless of the pathology, heart rate variability varies inversely with clinical severity and prognosis.

## Abstract (pt)

**Objetivo:**

Apresentar uma revisão sistemática do uso da monitorização do sistema nervoso autônomo como ferramenta de prognóstico, verificando a variabilidade da frequência cardíaca nas unidades de cuidados intensivos.

**Métodos:**

Revisão de literatura publicada até julho de 2016 na PubMed/MEDLINE de estudos realizados em unidades de cuidados intensivos, sobre a monitorização do sistema nervoso autônomo, por meio da análise da variabilidade da frequência cardíaca, como ferramenta de prognóstico - estudo da mortalidade. Foram utilizados os seguintes termos em inglês no campo de pesquisa: ("*autonomic nervous system*" OR "*heart rate variability*") AND ("*intensive care*" OR "*critical care*" OR "*emergency care*" OR "ICU") AND ("*prognosis*" OR "*prognoses*" OR "*mortality*").

**Resultados:**

A probabilidade de morte nos doentes aumentou com a diminuição da variabilidade da frequência cardíaca, estudada por meio da variância da frequência cardíaca, desacoplamento cardíaco, volatilidade da frequência cardíaca, *integer heart rate variability*, desvio padrão de todos os intervalos RR normais, raiz quadrada da média do quadrado das diferenças entre intervalos RR adjacentes, poder total, componente de baixa frequência, componente de muito baixa frequência, razão entre o componente de baixa frequência e o componente de alta frequência), razão entre expoentes fractais de curto e longo prazo, entropia de Shannon, entropia multiescalar e entropia aproximada.

**Conclusão:**

Nos doentes internados em unidades de cuidados intensivos, independentemente da patologia que motivou o internamento, a variabilidade da frequência cardíaca varia de forma inversa com a gravidade clínica e com o prognóstico.

## INTRODUCTION

Since the 1970s, with the introduction of the Swan-Ganz catheter,^(1)^ there has been significant progress in the capacity of invasive and non-invasive hemodynamic monitoring in intensive care units (ICU) and an improved understanding of the pathophysiological phenomena responsible for the hemodynamic instability of critical patients.

Despite these remarkable advances, there is no unanimity as to what therapeutic objectives should be achieved in patients with hemodynamic instability admitted to the ICU,^(2)^ for the time being maintaining an individual therapeutic attitude guided not by hemodynamic monitoring data but by the integration of the different variables that can be obtained using multiple monitoring methods.

This situation results from an overvaluation of our view of the cardiovascular system according to physics principles rather than a look at the capacity and adjustment of the real-time responses of critical patients to the pathophysiological changes induced by the disease and imposed by our therapeutic attitudes, either pharmacological or not. More important than the "normalization" of a given parameter is its temporal adjustment.

Recent studies^(3-5)^ have described several hemodynamic monitoring methods, from the most invasive, such as the Swan-Ganz catheter, to the less invasive, such as bioimpedance and bioreactance methods. However, although the autonomic nervous system (ANS) is responsible for the homeostasis of the cardiocirculatory system through the balance between the activity of the sympathetic and parasympathetic ANS, no reference is made to the monitoring of its activity and/or its balance in ICU patients.

**Figure 1:**
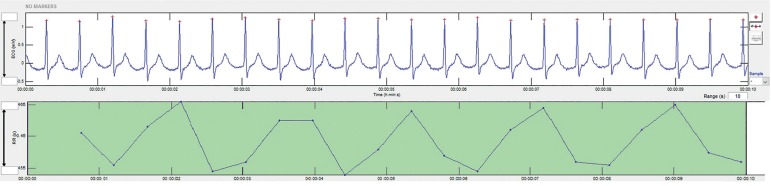
Ten-second cardiotocogram showing heart rate variability.

Heart rate variability (HRV) translates the oscillations in the duration of intervals between consecutive heart beats (NN intervals) (Figure 1) and is related to the influences of the ANS on the sinus node, translating the heart's capacity to respond to multiple physiological and environmental stimuli, such as breathing, physical exercise, hemodynamic and metabolic changes, orthostatism and responses to stress induced by diseases. Moreover, the study of HRV of the ANS is only possible in the presence of sinus rhythm.

The objective of this article is to present a systematic review of studies involving autonomic nervous system monitoring of adult patients admitted to the intensive care units by analyzing the association of multiple heart rate variability assessment measures with the hospitalization outcome. Prospective and retrospective randomized controlled or cohort studies were included.

## METHODS

In this systematic review, we used the checklist Preferred Reporting Items for Systematic Reviews and Meta-Analyses (PRISMA)^(6)^ as a guide to reach the standards accepted in systematic reviews.

The literature review of studies conducted in ICUs on ANS monitoring was conducted by searching all of the measures described for HRV analysis methods (Tables 1 and 2) as a prognostic tool (mortality study), published in or before July 2016 (inclusive) using the PubMed/MEDLINE database. The following English terms were entered in the search field, yielding 421 articles: ("autonomic nervous system" OR "heart rate variability") AND ("intensive care" OR "critical care" OR "emergency care" OR "ICU") AND ("prognosis" OR "prognoses" OR "mortality").

**Table 1:** Methods for the study of heart rate variability^(7,8,9)^

1. Linear methods - time domain
a. Statistical measures
i. SDNN - Standard deviation of all normal NN intervals
ii. SDANN - Standard deviation of the average normal NN interval calculated over 5-minute intervals
iii. SDNNi - Mean of the standard deviations of all normal NN calculated over 5-minute intervals
iv. rMSSD - Square root of the mean squared differences of successive normal NN intervals
v. SDSD - Standard deviation of differences between adjacent normal NN intervals
vi. NN50 - Number of pairs of adjacent normal NN intervals differing by more than 50 milliseconds
vii. pNN50 - Percentage of normal NN intervals differing by more than 50 milliseconds from the adjacent interval
b. Geometric measures
i. Triangular index
ii. TINN - Triangular interpolation of normal NN intervals histogram
iii. Differential index
iv. Logarithmic index
2. Linear methods - frequency domain
a. Long-term analysis (5 minutes)
i. Total power
ii. VLF - Very low frequency
iii. LF - Low frequency
iv. LFn - Low frequency in normalized units
v. HF - High frequency
vi. HFn - High frequency in normalized units
vii. LF/HF - Low frequency/high frequency ratio
b. Long-term analysis (24 hours)
i. Total power
ii. ULF - Ultra low frequency
iii. VLF - Very low frequency
iv. LF - Low frequency
v. HF - High frequency
vi. α - Slope of the linear interpolation of the spectrum in a logarithmic scale
3. Time-frequency analysis methods
a. Time-varying parametric models
i. Autoregression models
b. Non-parametric methods
i. Short-time Fourier transform (STFT)
ii. Wavelet transform (WT)
iii. Hilbert-Huang transform
iv. Wigner-Ville transform
4. Non-linear methods
a. Detrended fluctuation analysis (total DTA, α1, α2 and α1/α2)
b. Correlation function
c. Hurst exponent
d. Fractal dimension
e. Lyapunov exponent
f. Sample entropy
g. Multiscale entropy
h. Approximate entropy (ApEn)
i. Shannon entropy

**Table 2:** Definition of measures for the study of heart rate variability in the time domain^(7)^

Measure	Unit	Definition
SDNN	ms	Standard deviation of all normal NN intervals
SDNNi	ms	Standard deviation of NN calculated over 5-minute intervals
SDANN	ms	Standard deviation of the average NN interval
rMSSD	ms	Root mean square of the successive NN interval difference
pNN50	%	Normal-to-normal NN intervals whose difference exceeds 50 milliseconds

After applying the filters to limit the studies to those involving humans aged over 19 years, without language restriction, 193 articles were excluded.

After reading the abstracts of the 228 selected studies, 180 articles were excluded: 11 reported the monitoring of pediatric patients, 16 were conducted outside the intensive care setting, 119 were not related to ANS monitoring, four did not analyze HRV, 28 did not focus on prognosis and two were review studies.

The 48 articles selected were grouped and cataloged in EndNote^®^ and were read in full. Afterwards, 32 articles were excluded: 21 because they were not studies of ICU patients (11 were performed in the Emergency Department, five in the prehospital setting, two in the Cardiothoracic Surgery Service and two in the Cardiology Service, and one study was conducted during the anesthetic period) and 11 because they did not report mortality data.

**Figure 2:**
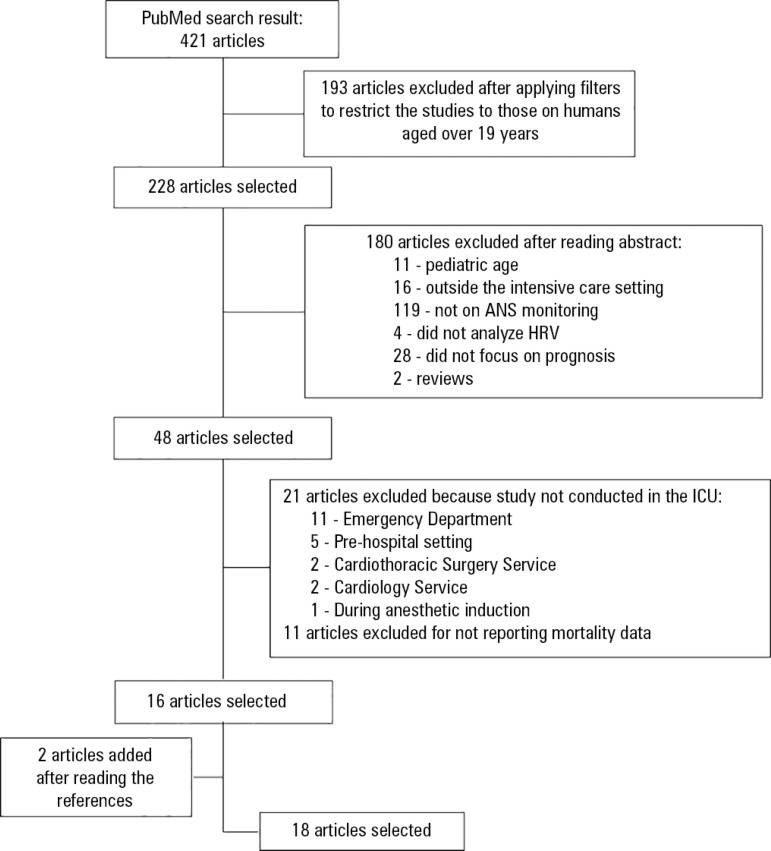
Article selection protocol.^(6)^ HRV - heart rate variability; ICU - intensive care unit.

The references of the 16 selected articles were reviewed, and whenever there was reference to a new study, that study was evaluated; at the end of the review process, 18 articles were selected (Figure 2).

The quality of evidence for each selected study was assessed using the Methodological Index for Non-Randomized Studies (MINORS) tool.^(10)^

The article review (data extraction and quality of evidence) was conducted by one author, with the information later independently verified by two others.

Table 3 shows the characteristics of the selected studies.

**Table 3:** Characteristics of the selected studies

Author	Characteristics	Evaluated outcomes	Results	MINORS (score/total)
Pfeifer et al.^(11)^	Prospective cohort study Patients admitted to the ICU after cardiac arrest, subjected to therapeutic hypothermia N = 18	28-day mortality	There was a more pronounced reduction in HRV immediately after the rewarming phase in patients who died compared with survivors (SDNN 10.9 *versus* 40.2, Shannon entropy 2.2 *versus* 3.7)	15/24
Riordan et al.^(12)^	Retrospective cohort study Multiple trauma patients admitted to the ICU N = 2,178	Risk of death in the subgroups based on trauma location and mechanism and on probability of survival	Decreased MSE was significantly associated with increased mortality, being an independent factor of probability of survival in the multivariate analysis, with OR 0.87 - 0.94; the difference in median HR of MSE between survivors and non-survivors was highest (15.9 *versus* 5.9) when the primary trauma mechanism was penetrating	10/24
Kahraman et al.^(13)^	Prospective cohort study Patients admitted to the ICU with head trauma with Glasgow coma scale score < 9 and need for ICP monitoring N = 25	Capacity to predict intracranial hypertension, cerebral hypoperfusion, in-hospital mortality or functional outcome	HRVi* can predict in-hospital mortality, with a sensitivity of 67% and a specificity of 91-100%	15/24
Mowery et al.^(14)^	Retrospective cohort study Patients with head trauma and ICP monitoring N = 145	Intracranial hypertension and mortality	There is a relationship between percentage of ICP rise and cardiac decoupling with mortality. Each percentage increase had an increased risk of death of 1.04 and 1.03, respectively	15/24
Norris et al.^(15)^	Retrospective cohort study Trauma patients admitted to the ICU N = 285	In-hospital mortality	There was a decrease in HRV (increase in HRVi*), OR 1.04 ± 0.01 and MSE OR 0.88 ± 0.03, in deceased patients	12/24
Papaioannou et al.^(16)^	Prospective cohort study Head trauma N = 20	Neurological dysfunction ICU mortality	It was associated with increased mortality, reduced heart rate variability, reduced baroreflex sensitivity and sustained LF/HF ratio reduction	17/24
Norris et al.^(17)^	Retrospective cohort study Trauma patients admitted to the ICU N = 2,088	Mortality	Cardiac decoupling was associated with increased mortality OR 1.035 - 1.052	13/24
Grogan et al.^(18)^	Retrospective cohort study Trauma patients admitted to the ICU N = 923	ICU mortality	Patients with loss of heart rate volatility during the first 24 hours of hospitalization have a higher probability of death	10/24
Rapenne et al.^(19)^	Prospective cohort study Severe head trauma N = 20	Brain death Neurological recovery (Glasgow coma scale)	On the first post-trauma day, an increase in the parasympathetic tone (rMSSD and TP) may be associated with imminent brain death	17/24
Winchell et al.^(20)^	Retrospective cohort study Patients with severe head trauma N = 80	Primary: in-hospital mortality and probability of discharge to the home Secondary: CPP and ICP	Low HRV was associated with increased mortality; patients with a predominance of sympathetic activity and with a low HF/LF ratio had improved survival	16/24
Brown et al.^(21)^	Prospective cohort study Patients admitted to the ICU with severe sepsis or septic shock N = 48	Primary outcome: suspension of vasoactive amines within the first 24 hours of ICU admission Secondary outcome: 28-day mortality	The ratio between short- and long-term fractal exponents was associated with 28-day mortality; all patients who died had ratios < 0.75	18/24
Schmidt et al.^(22)^	Prospective cohort study Patients with multiple organ dysfunction syndrome N = 90	Analysis of survival at 180 and 365 days	lnVLF† with a cutoff point of 3.9 was a strong predictor of 28-day and 2-month mortality in patients with multiple organ dysfunction syndrome	18/24
Schmidt et al.^(23)^	Prospective cohort study Patients with multiple dysfunction syndrome N = 90	28-day mortality	lnVLF† with a cut-off point of 3.9 was a strong predictor of 28-day mortality	20/24
Gujjar et al.^(24)^	Prospective cohort study Acute stroke N = 25	ICU mortality	LFn was an independent predictor of survival, with a regression coefficient of -6.73 and an OR of 0.002	19/24
Haji-Michael et al.^(25)^	Prospective cohort study Neurosurgical patients with Glasgow coma scale score < 13 N = 29	3-month outcome	Patients who died had decreased HRV, LF/HF ratio and baroreflex sensitivity	18/24
Papaioannou et al.^(26)^	Prospective cohort study General ICU population N = 53	ICU mortality	The minimum ApEn value correlated with mortality (r = 0.41; p = 0.01)	16/24
Yien et al.^(27)^	Prospective cohort study General population admitted for noncardiac causes N = 52	Mortality	Deceased patients had decreased VLF and LF band power	16/24
Winchell et al.^(28)^	Prospective cohort study General ICU population N = 742	Mortality	The relative risk of death in patients with low HRV was 7.4, with an increased HF/LF ratio of 4.55	19/24

MINORS - Methodological Index for Non-Randomized Studies; ICU - intensive care unit; HRV - heart rate variability; MSE - multiscale entropy; OR - odds ratio; HR - hazard ratio; HRVi - integer heart rate variability; ICP - intracranial pressure; LF/HF - ratio between the low frequency component and the high frequency component; CPP - cerebral perfusion pressure; TP - total power.

* Calculation of the standard deviation of the electrocardiogram signal collected every 1-4 seconds during a 5-minute interval;

† natural logarithm of VLF.

## RESULTS

The 18 selected studies are presented in table 3. The type of study, study population, number of patients included, HRV variables studied in the ANS monitoring, most relevant conclusions and quality of evidence were also analyzed.

All studies reviewed were cohort, prospective or retrospective studies. The sample size was very heterogeneous, ranging from 18^(11)^ to 2,178^(12)^ patients; the sample size was not previously calculated in any study. The most studied pathology was trauma, mainly of the head, with a total of nine studies,^(12-20)^ and with the same number of studies on patients with severe sepsis and septic shock,^(21)^ multiple dysfunction syndrome,^(22,23)^ patients undergoing therapeutic hypothermia after cardiac arrest,^(11)^ with stroke^(24)^ and neurosurgical patients;^(25)^ three studies focused on the general population admitted to the ICU, without discriminating the reason for admission. The conclusions of all of the studies were obtained by comparing the groups according to the outcome evaluated, namely, mortality.

The results presented included increases in mortality associated with reduction in HRV (entropy 0.65 ± 0.24 *versus* 0.84 ± 0.26; p < 0.05), reduction in the baroreflex (transfer function 0.43 ± 29 *versus* 1.11 ± 0.74; p < 0.05) and a sustained reduction of the low frequency/high frequency ratio (LF/HF ratio 0.22 ± 0.29 *versus* 0.62 ± 28; p < 0.01);^(16)^ reductions in HRV, with odds ratios (ORs) of 1.03^(14)^ and of 1.035 - 1.052;^(17)^ loss of heart rate volatility during the first 24 hours of hospitalization, translated as a coefficient of 0.05 in the logistic regression model (95% confidence interval [95% CI] 1.033 - 1.071);^(18)^ integer heart rate variability (HRVi) with a sensitivity of 67% and a specificity of 91 - 100% to predict the mortality rate^(13)^ or OR of 1.04;^(15)^ and reduction in HRV in patients admitted to the ICU after cardiac arrest and undergoing therapeutic hypothermia, with a standard deviation of all normal NN intervals of 10.9 ± 4.1 *versus* 40.2 ± 19.5 (p = 0.01) and a Shannon entropy of 2.2 ± 0.4 *versus* 3.7 ± 0.6 (p = 0.008) for deceased *versus* surviving patients in the rewarming period. Concordant results were observed in the pre-hypothermia period.^(11)^ There was also an increase in the parasympathetic tone as measured by the square root of the mean squared differences of successive intervals (rMSSD) (34.07 ± 6.54 *versus* 15.51 ± 3.90; p = 0.01) in patients with severe head injury;^(19)^ decreased power in the low frequency band (low frequency in standard units in patients with severe stroke 18.90 ± 1.36 *versus* 49.66 ± 2.10; p = 0.02; in the general population p < 0.05 with Scheffé analysis);^(24,27)^ decreased natural logarithm of the very low frequency band (lnVLF £ 3.9 with OR 2.9; in the general population p < 0.05 with Scheffé analysis);^(22,23,27,28)^ and decreased ratio of short- to long-term fractal exponents; all patients admitted to the ICU with severe sepsis or septic shock who died had a ratio of < 0.75 (p = 0.04).^(21)^ The following were also found: decreased multiscale entropy in trauma patients (8.9 *versus* 16.6; p < 0.0001; 7.5 *versus* 11.2; p < 0.001 in patients with survival probabilities < 0.25; 7.7 *versus* 12.8; p < 0.01 for patients with survival probabilities of 0.25 to 0.50; 9.4 *versus* 15.0; p < 0.001 for patients with survival probabilities of 0.50 to 0.75; 9.9 *versus* 16.1; and p < 0.001 among those with survival probabilities ³ 0.75).^(12,15)^ Decreased approximate entropy (mean ApEn 0.53 ± 0.25 *versus* 0.62 ± 0.28; p = 0.04; minimum ApEn 0.24 ± 0.23 *versus* 0.48 ± 0.23; p = 0.01) with a Pearson coefficient of 0.41 (p = 0.01) was also found.^(26)^

Thus, these studies showed that, in patients admitted to the ICU, regardless of the pathology that led to hospitalization, HRV varied inversely with clinical severity and prognosis.^(29)^

## DISCUSSION

The control of the cardiovascular system is ensured by the balance between the activity of the sympathetic ANS, which enervates the entire myocardium, and the parasympathetic ANS, which enervates the sinus node, the atrial myocardium and the atrioventricular node.^(30)^ The influence of the ANS on the heart depends on the information it receives from the baroreceptors, chemoreceptors, atrial receptors, ventricular receptors, changes in the respiratory system, vasomotor system, renin-angiotensin-aldosterone system and thermoregulatory system.^(31)^ All of these influences condition the HRV, and the standards for its measurement, physiological interpretation and applicability were published in 1996.^(7)^

The HRV can be analyzed using different methods, with linear methods being the most used in clinical practice.

The time domain is analyzed using various measures and reflects the variation in the duration of NN intervals resulting from the depolarization of the sinus node.

Analysis of the frequency domain decomposes the HRV into the high frequency band, ranging between 0.15 and 0.4 Hz, which corresponds to the respiratory modulation, translating the parasympathetic activity; the low frequency band, ranging between 0.04 and 0.15 Hz, which corresponds to sympathetic and parasympathetic activity; the very low frequency band, ranging between 0.003 and 0.04 Hz, which reflects the thermoregulation cycles; and ultra low frequency components, with variations below 0.003 Hz, modulated by the circadian rhythm and neuroendocrine axes.

The inverse relationship enters the very low frequency band, and the prognosis was first described in the 1960s,^(32)^ when it was observed that NN interval reduction preceded fetal distress.

The first study conducted in the ICU was published in 1996 and concluded that HRV reduction was related to increased mortality.^(28)^ Since then, all studies conducted in the ICU have almost exclusively focused on the evaluation of HRV, which varies inversely with clinical severity and prognosis.^(29)^

Examples of clinical conditions in which HRV is predictive of patient survival include diabetes,^(33)^ cancer,^(34)^ heart failure,^(35)^ acute myocardial infarction,^(36)^ stroke,^(37)^ epilepsy,^(38)^ Parkinson's disease^(39)^ and kidney failure,^(40)^ among others.

In patients admitted to the ICU, in addition to being used as a prognostic tool, HRV has also been described as a screening tool for multiple trauma patients,^(41)^ as a tool for individual monitoring of organ dysfunction,^(42)^ as a non-invasive tool for pain monitoring^(43)^ and as an independent predictor factor for the prolongation of hospital stay in patients undergoing heart surgery^(44)^ and has been used as a tool for successful extubation decision-making.^(45,46)^

Some limitations were identified in the studies reviewed. There is no uniformity in the variables studied for HRV assessment, although the studies are concordant in the conclusions presented; furthermore, the quality of the evidence is low, due mainly to the sampled studies being cohort studies.

## CONCLUSION

Heart rate variability occurs inversely to clinical severity and prognosis. The difficulty of introducing autonomic nervous system monitoring in the daily practice of intensive care units is due to the limitation of its use as a prognostic tool and, above all, to the difficulties involved in continuous and dynamic monitoring and in the interpretation and applicability of its results.

Successful implementation depends on heart rate variability monitoring going from a prognostic tool to a real-time monitoring instrument in order to be useful in therapeutic guidance; for example, as a guide for fluid therapy through analysis of the high frequency component and for treatment with vasoactive amines through analysis of the low frequency/high frequency ratio.
